# Sleep Patterns of Premedical Undergraduate Students: Pilot Study and Protocol Evaluation

**DOI:** 10.2196/45910

**Published:** 2024-02-02

**Authors:** Gargi Rajput, Andy Gao, Tzu-Chun Wu, Ching-Tzu Tsai, Jennifer Molano, Danny T Y Wu

**Affiliations:** 1 Department of Biomedical Informatics College of Medicine University of Cincinnati Cincinnati, OH United States; 2 Medical Sciences Baccalaureate Program College of Medicine University of Cincinnati Cincinnati, OH United States; 3 School of Design University of Cincinnati Cincinnati, OH United States; 4 Department of Neurology and Rehabilitation Medicine College of Medicine University of Cincinnati Cincinnati, OH United States

**Keywords:** patient-generated health data, Fitbit wearables, sleep quality, premedical college students, sleep, sleep hygiene, student, colleges, university, postsecondary, higher education, survey, sleep pattern, medical student, adolescence, behavior change

## Abstract

**Background:**

Poor sleep hygiene persists in college students today, despite its heavy implications on adolescent development and academic performance. Although sleep patterns in undergraduates have been broadly investigated, no study has exclusively assessed the sleep patterns of premedical undergraduate students. A gap also exists in the knowledge of how students perceive their sleep patterns compared to their actual sleep patterns.

**Objective:**

This study aims to address 2 research questions: What are the sleep patterns of premedical undergraduate students? Would the proposed study protocol be feasible to examine the perception of sleep quality and promote sleep behavioral changes in premedical undergraduate students?

**Methods:**

An anonymous survey was conducted with premedical students in the Medical Science Baccalaureate program at an R1: doctoral university in the Midwest United States to investigate their sleep habits and understand their demographics. The survey consisted of both Pittsburg Sleep Quality Index (PSQI) questionnaire items (1-9) and participant demographic questions. To examine the proposed protocol feasibility, we recruited 5 students from the survey pool for addressing the perception of sleep quality and changes. These participants followed a 2-week protocol wearing Fitbit Inspire 2 watches and underwent preassessments, midassessments, and postassessments. Participants completed daily reflections and semistructured interviews along with PSQI questionnaires during assessments.

**Results:**

According to 103 survey responses, premedical students slept an average of 7.1 hours per night. Only a quarter (26/103) of the participants experienced good sleep quality (PSQI<5), although there was no significant difference (*P*=.11) in the proportions of good (PSQI<5) versus poor sleepers (PSQI≥5) across cohorts. When students perceived no problem at all in their sleep quality, 50% (14/28) of them actually had poor sleep quality. Among the larger proportion of students who perceived sleep quality as only a slight problem, 26% (11/43) of them presented poor sleep quality. High stress levels were associated with poor sleep quality. This study reveals Fitbit as a beneficial tool in raising sleep awareness. Participants highlighted Fitbit elements that aid in comprehension such as being able to visualize their sleep stage breakdown and receive an overview of their sleep pattern by simply looking at their Fitbit sleep scores. In terms of protocol evaluation, participants believed that assessments were conducted within the expected duration, and they did not have a strong opinion about the frequency of survey administration. However, Fitbit was found to provide notable variation daily, leading to missing data. Moreover, the Fitbit app’s feature description was vague and could lead to confusion.

**Conclusions:**

Poor sleep quality experienced by unaware premedical students points to a need for raising sleep awareness and developing effective interventions. Future work should refine our study protocol based on lessons learned and health behavior theories and use Fitbit as an informatics solution to promote healthy sleep behaviors.

## Introduction

Sleep health has been recognized as an unresolved public health concern since 2006 [1]. Although sleep plays a vital role in adolescent health and development, poor sleep patterns continue to be associated with college students. A 2015 study showed that 70%-96% of college students sleep less than the recommended 8 hours on weeknights [2,3]. With the heavy prevalence of sleep deprivation, it is important to recognize the detrimental effects it can have. Starting with cognitive processing, poor sleep has been shown to have negative effects on working memory, attention span, and speed of processing [4]. With poor sleep, our bodies can also experience impaired immune function, more susceptibility to illnesses, increased risk of stress, and decreased physical functions [5]. In addition, emotional dysregulation and lower levels of subjective well-being can be observed [6]. As a result, students experience a consequential effect on their academic performance [7]. It is therefore imperative to understand and discuss the sleep patterns of adolescents, especially college students, in order to aid them with tools that can help avoid these consequences.

Additionally, it is possible that students who may appear to receive an adequate amount of sleep can experience poor sleep due to bad sleep quality. Sleep quality refers to both quantitative aspects of sleep such as duration, sleep latency, and number of arousals, and qualitative aspects such as depth and restfulness of sleep according to a 1998 Psychiatry and Research publication [8]. Poor sleep quality refers to the struggle of falling asleep or maintaining sleep. A recent 2022 study clarifies this definition by adding the component of individuals’ self-satisfaction with their sleep as another part of sleep quality [9]. A previous survey on college undergraduates found that only 34.1% of students displayed good sleep quality based on Pittsburg Sleep Quality Index (PSQI) scores [10]. The PSQI is a self-rated questionnaire that serves as a standard tool for measuring sleep quality [8]. The responses to questionnaire items allow researchers to determine a global PSQI score, which helps distinguish between good-quality and poor-quality sleepers. A PSQI score smaller than 5 (<5) signifies good sleep quality, whereas a PSQI score equal to or larger than 5 (≥5) indicates poor sleep quality. However, to diagnostically study sleep, polysomnography (PSG) can be conducted, which involves recording brainwaves, oxygen level in the blood, respiration rate, and physical movements to diagnose sleep disorders [11]. PSG is used to understand the sleep patterns of patients and why there might be disruptions [12].

A recent study on the sleep quality of college students explored the determinants of sleep quality [13], interventions for improving sleep quality, overall demographics of sleep patterns, and associations of poor sleep quality with health problems. Some studies [7,14] have approached the understanding of sleep through a comparison of different college majors. One such major or a group of students that is of interest to us are students who are premedicine (premedical) majors or undergraduate students on the prehealth track. However, there is limited exploration looking specifically at the sleep patterns of premedical undergraduate students (premedical students hereafter). Previous research in this area has largely been centered on the sleep quality of students who are in a medical school or graduate medical education program and focused on sleep-related disorders among them [15-19]. Numerous studies have measured the sleep patterns of medical students but have not focused on understanding the patterns [20-33]. To date, only 1 study has tested medical students on their sleep knowledge and has found a disconnect between their understanding of sleep facts and actual sleep quality [34]. This led to the focus of our study on the sleep quality perception of premedical students.

A student’s perception of his or her sleep habits can give us insights into whether perception influences sleep behaviors and quality. Many prior studies [35-38] have looked at sleep state misperception. This concept discusses how there is a disconnect between the amount of sleep people think they get versus their actual duration of sleep. Research on sleep state misperception has primarily been conducted on people dealing with varying mental health conditions or sleep disorders [35-38]. In this manner, our research focused on the effect of sleep quality perception on sleep patterns, which considers overall sleep quality rather than just duration, in a relatively healthy population comprised of premedical students. Literature shows that in medical students, perception of stress can have an impact on their well-being [39], while another study has confirmed that there is a positive effect of education on sleep in undergraduates [40]. However, there is a gap in the literature in terms of understanding how premedical students perceive their sleep versus their actual sleep patterns. Although one study comes close to answering this question by examining the beliefs of sleep hygiene in medical students compared to their actual sleep practices [41], the behavior of premedical undergraduates is yet to be explored and understood.

Current validation studies suggest that while PSG remains the gold standard, Fitbit is capable of monitoring various sleep parameters that do not significantly deviate from PSG measurements [42-44]. The overall objective of our research was to understand the sleep patterns of premedical students and create interventions to promote positive behavioral changes. Our pilot study focuses on answering the following 2 research questions (RQs). What are the sleep patterns of premedical undergraduate students (RQ1)? Would the study protocol be feasible to examine the perception of sleep quality and promote sleep behavioral changes in premedical undergraduate students (RQ2)?

## Methods

### Setting

This study was conducted with premedical students from a Medical Sciences Baccalaureate Program (MSBP) at the University of Cincinnati, an R1: doctoral university and an undergraduate College of Medicine in the Midwest United States. The MSBP is a program designed to help premedical students prepare for professional graduate schools in the health care field. The 4-year program consists of 376 students as of October 2022.

### Study Design

**Figure 1:**
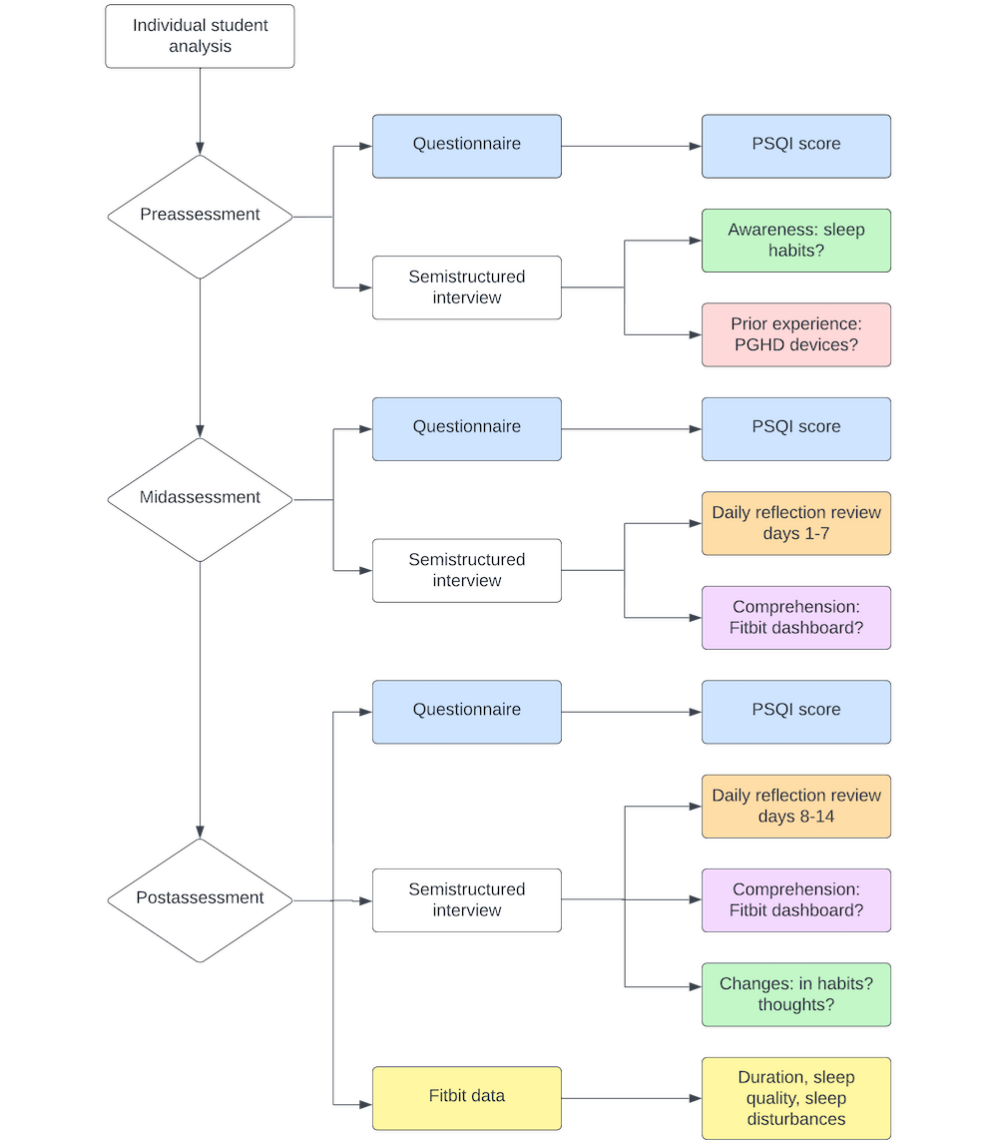
Flowchart of the study protocol tested on 5 premedical participants during the 2-week pilot study with Fitbit Inspire 2. Week 0 started with preassessment, conducted in person at the University of Cincinnati Medical Sciences building. Week 1 midassessment and Week 2 postassessment were conducted virtually through Microsoft Teams meeting. The data collection was conducted in 2 cycles. Participants 1, 2, and 3 began the 2-week protocol in the last week of July 2022 and finished in the second week of August 2022. Participants 4 and 5 began the 2-week protocol in the first week of September 2022 and finished in the third week of September 2022. All of the participants had varying start dates during this time frame, but each participant was available for 10 weekdays and 4 weekend days during the duration of the 2-week data collection. PGHD: patient-generated health data; PSQI: Pittsburg Sleep Quality Index.

To address RQ1, a “Well-being and Sleep Survey” (the well-being survey hereafter) was distributed to collect subjective sleep patterns. To address RQ2, a small group of participants (MSBP premedical students) were recruited to evaluate whether the study protocol (Figure 1) was feasible and could generate valid data for analysis. Specifically, the participants put on the Fitbit Inspire 2 wearable watch to monitor their sleep patterns. These participants were then invited to a semistructured interview to understand their Fitbit app experience. As shown in Figure 1, during the 2-week pilot study period, participants progressed through preassessment (week 0), midassessment (week 1), and postassessment (week 2) periods. The well-being survey was filled out by participants at all 3 fixed time points. During week 0, a semistructured interview was conducted assessing participants about their typical sleep habits and their previous experience with wearable devices. Participants at this point were also prompted to fill out a form daily, reflecting their thoughts regarding the Fitbit sleep score they received the night before. On week 1, a midassessment was performed in which the participants were asked to speak about their previous week’s experience with the aid of the daily reflections they had previously filled for days 1-7. In order to prompt the participants, researchers displayed a line chart of the 7 responses gathered from participants’ daily forms to project their sleep score trends over the week. Researchers also listed all participants’ free-text responses so that they could elaborate specifically about their sleep corresponding to certain dates. Lastly, in week 2, participants reviewed the daily reflections for days 8-14, which was followed by a semistructured interview.

### Ethics Approval

This study was reviewed and approved by the University of Cincinnati Institutional Review Board (approval 2021-1119) and determined as not human subject research due to its collection on de-identified data and the focus on quality improvement. This institutional review board determination resulted in the avoidance of collecting detailed student demographics such as sex and age and the deidentification of all participant information in the data.

### Participant Recruitment

The survey participants were recruited on a voluntary basis via email and GroupMe messages through the research team’s professional and personal network. The small group of participants for the protocol evaluation was selected from the survey participants as a convenience sample. This study was expected to target 100 survey participants and 5 protocol evaluation participants.

### Data Collection

The well-being survey was distributed among all cohorts (years 1, 2, 3, and 4 students) of the MSBP program (N=376). The survey consisted of PSQI items (1-9) adjusted to items (1-11) to accommodate the web-based survey format due to some combined line items in the original paper format, requiring different lines in the web-based survey. The response components combined to calculate a global PSQI score (with a Cronbach α of .83) [8], which suggests a high degree of internal consistency. These items were followed by a few questions assessing students’ demographics and their perception of their sleep quality. The well-being survey was created in Microsoft forms with survey questions as listed in Table 1. For the protocol evaluation, 5 students were selected from the well-being survey pool. In addition to the sleep patterns collected from the self-reported PSQI survey and the objective Fitbit wearables, these 5 participants’ feedback on the protocol was collected through semistructured interviews. The interview was recorded and conducted on a virtual platform and further analyzed thematically [45]. For the Fitbit data collection, 5 Fitbit Inspire 2 watches were deployed—one for each participant. Fitbit data were collected by linking the watch to the participant’s Fitbit web account. During the semistructured interview in the midassessment, the participants were instructed to log into their account on Fitbit.com. Next, they were asked to navigate to “Settings,” select “Data Export,” and then click on “Request Data.” This generated an email request to the participant to “Confirm Export Request.” Finally, after confirmation, the participant received a downloaded data zip file with JSON files containing complete archive data. The participants were then prompted to email the zip file to the researcher. This process was again repeated during the postassessment. Table 1 identifies each question sequentially, as it was presented in the web-based Microsoft form, as well as categorizes it as either a PSQI question or a demographic question. PSQI questions are directly derived from PSQI questionnaire items (1-9), which are adjusted to items 1-11 in our survey to accommodate the web-based format as compared to the original PSQI questionnaire paper format. The description lists exactly how the questions were asked to premedical MSBP students. The response type specifies whether survey participants responded to the question by inputting free text or selecting their answers on the Likert scale or multiple choice.

**Table 1:** Well-being survey questions that were administered to 376 students in the Medical Sciences Baccalaureate program at the University of Cincinnati.

Sequence	Category	Description	Response type/scale
1	PSQI^a^	During Week 01, when have you usually gone to bed at night?	Free text
2	PSQI	During Week 01, how long (in minutes) has it usually taken you to fall asleep each night?	Free text
3	PSQI	During Week 01, when have you usually gotten up in the morning?	Free text
4	PSQI	During Week 01, how many hours of actual sleep did you get at night? (This may be different from the number of hours you spend in bed)	Free text
5	PSQI	During Week 01, how often have you had trouble sleeping because you... a....cannot get to sleep within 30 min b....wake up in the middle of the night or early morning c...have to get up to use the bathroom d...cannot breathe comfortably e...cough or snore loudly f...feel too cold g...feel too hot h...had bad dreams i...have pain	Likert
6	PSQI	If you have other reasons for having trouble sleeping, please describe...	Free text
7	PSQI	How often during Week 01 have you had trouble sleeping because of Q6?	Likert
8	PSQI	How often during Week 01 have you had trouble sleeping because of this?	Likert
9	PSQI	During Week 01, how often have you taken medicine (prescribed or “over the counter”) to help you sleep?	Likert
10	PSQI	During Week 01, how often have you had trouble staying awake while driving, eating meals, or engaging in social activity?	Likert
11	PSQI	During Week 01, how much of a problem has it been for you to keep up enough enthusiasm to get things done?	Likert
12	Demographics	Year in MSBP^b^ program?	Multiple choice
13	Demographics	Sleep quality: Do you have concerns about your sleep quality? How much role does stress play in your sleep quality?	Likert
14	Demographics	Have you ever seen a sleep specialist for sleep problems?	Likert
15	Demographics	Have you ever worn a wearable device to track your sleep?	Multiple choice
16	Demographics	Are you interested in joining a Fitbit usability study?	Multiple choice
17	Demographics	If yes to Q16, then enter your email.	Free text

^a^PSQI: Pittsburg Sleep Quality Index.

^b^MSBP: Medical Sciences Baccalaureate Program.

### Survey Data Analysis

After MSBP premedical students completed the well-being survey, the responses were analyzed as follows. First, the PSQI items (questions 1-11) were scored based on PSQI scoring guidelines. Then, a global PSQI score was assigned to each survey participant. Next, 3 analyses were made: (1) comparing student distributions with good PSQI scores (<5) versus poor PSQI scores (≥5) categorized by each cohort, (2) detecting the consistency between the perception of sleep quality and the PSQI score levels, and (3) finding the PSQI score differences on the perception of stress effect levels. Statistical analyses were performed to test the distribution homogeneity of each cohort using the chi-square test. The consistency between the perception of sleep quality and PSQI score will be tested using Cohen κ by grouping PSQI scores into corresponding sleep quality levels. For comparing the global PSQI score differences between stress effect levels, global PSQI scores were first treated as a continuous variable and the equality of medians on each group was tested using the Kruskal-Wallis test (nonparametric equivalent test of 1-way analysis of variance) to show any statistical difference. Furthermore, the post hoc Dwass-Steel-Critchlow-Fligner multiple comparisons test was applied to show the pairwise comparisons if at least one group median was significantly different from others.

### Protocol Evaluation

The protocol evaluation was 2-fold. First, each assessment had an expected duration to avoid overwhelming the participants. The expected durations of the preassessments, midassessments, and postassessments were 30 minutes, 30 minutes, and 60 minutes, respectively. The postassessment was longer due to the semistructured interview. Second, the semistructured interview collected the participants’ feedback on the study protocol in 4 areas: (1) duration of the 3 (pre, mid, and post) assessments, (2) frequency of administering the well-being surveys, (3) completeness of Fitbit data, and (4) other suggestions. An additional analysis was conducted to assess the degree to which Fitbit data aligns with the self-reported data obtained through surveys by using Pearson correlation coefficients where >|0.7| is strong correlation, |0.3|-|0.7| is moderate correlation, and <|0.7| is poor correlation (of note, the vertical lines present the absolute value sign).

## Results

### Survey Responses and Analysis Results

**Figure 2:**
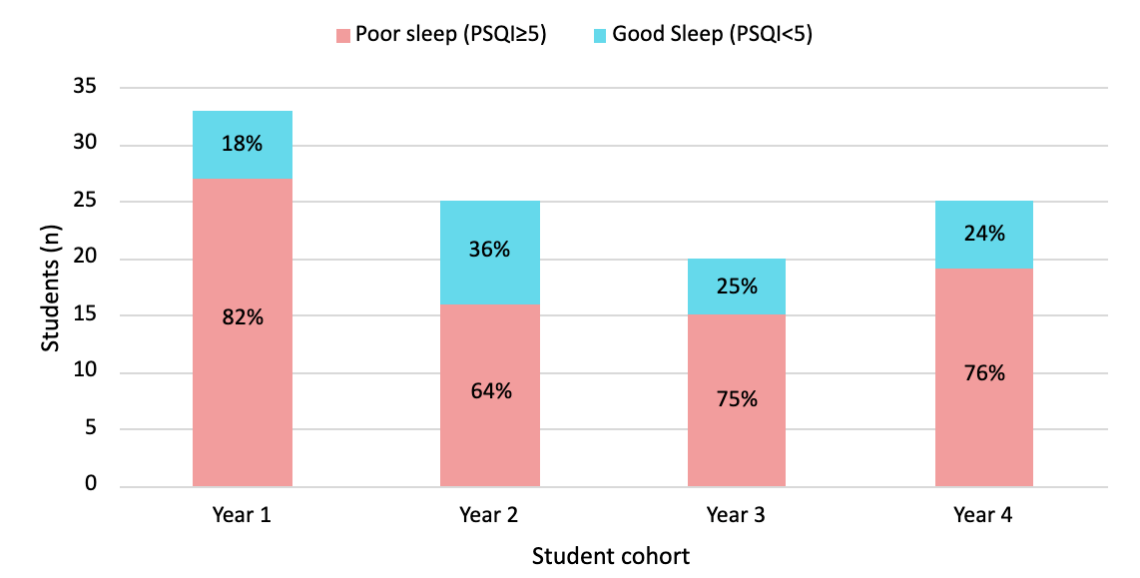
Stacked bar chart display of premedical student responses based on good sleep (global PSQI<5) and poor sleep (global PSQI≥5) with percentages within different medical sciences baccalaureate program cohorts. PSQI: Pittsburg Sleep Quality Index.

The survey responses (n=103) demonstrated that premedical MSBP students sleep an average of 7.1 hours each night with 81.3% habitual sleep efficiency (average hours slept versus hours spent in bed, as defined in PSQI). Those who experienced trouble sleeping commonly expressed reasons such as not being able to sleep within 30 minutes, waking up in the middle of the night or early morning, anxiety, stress, and a restless mind. Among the cohorts (Figure 2), no significant difference was found in the median PSQI scores as indicated by the Kruskal-Wallis test at a .05 significant level (*P*=.11). Similarly, the proportion of students who had good versus poor sleep was not significantly different as indicated by the chi-square test (*P*=.48). This concludes that the distributions have no significant differences in the good or bad PSQI scores among the student cohorts. It is worth noting that the response rates of the survey for the first-, second-, third-, and fourth-year cohorts were 38% (33/88), 23.4% (25/107), 23% (20/86), and 26% (25/95), respectively. Further, the same analysis was applied to the categorical variables collected in the survey data (Multimedia Appendix 1), including 2 non-PSQI variables (perceived sleep quality and perceived stress). The only significant difference was that the first-year students tended to take medicines to help themselves to sleep unlike the other cohorts (*P*=.03). However, there was no significant difference between the low frequency group (less than once a week and no medicine) and the high frequency group (once a week or more). To demonstrate the consistency between the perceived sleep quality and the PSQI scores, we assumed that students who perceived “no problem at all” will receive PSQI global scores <5, those who perceived “only a very slight problem” will receive PSQI global scores of 5-7, those who perceived “somewhat of a problem” will receive PSQI global scores of 8-10, and those who perceived “a very big problem” will receive PSQI global scores ≥ 11. The Cohen κ (with 1 being a perfect consistency) showed that participants’ perceived sleep quality only had a slight consistency with the PSQI scores (κ=0.19). Table 2 shows the contingency of these 2 variables with an overpositive tendency from participants, that is, the bigger the problem the participants perceived in their sleep quality, the poorer the PSQI score they would receive. However, the table shows some inconsistencies. When participants perceived no problem at all in their sleep quality, 50% (14/28) of them actually had a nonoptimal PSQI score. When the participants thought there was only a very slight problem in their sleep, about a quarter of them (11/43, 26%) had a poor PSQI score. When the participants appeared to recognize that they had somewhat of a problem (or a big one) in their sleep, the majority of them had nonoptimal PSQI scores.

**Figure 3:**
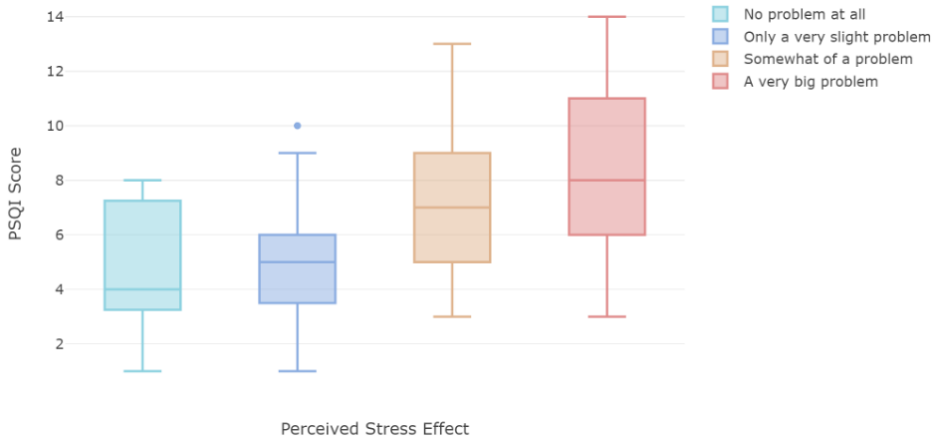
Boxplot of perceived stress effect on sleep by students compared to their global Pittsburg Sleep Quality Index scores. PSQI: Pittsburg Sleep Quality Index.

Finally, the comparisons between perceived stress impact and median global PSQI scores in each perceived stress level were analyzed (Figure 3). The Kruskal-Wallis test shows there is at least one median that is significantly different (*P*<.001) from others at the .05 significant level. The post hoc Dwass-Steel-Critchlow-Fligner multiple comparisons was applied and showed that the students who perceived “a very big problem” on the stress effect had significantly greater PSQI scores than the students who perceived “no problem at all” (*P*=.048) and “only a very slight problem” (*P*<.001). Similarly, for the students who perceived “somewhat of a problem,” the stress effect was significantly greater than that in students who perceived “only a very slight problem” (*P*=.001) but not significantly different from the “no problem at all” stress level (Table 3). This indicates that participants’ perceived stress effects have high correlation with their PSQI scores, but it may not be the only effect at play.

**Table 2:** The contingency table of Medical Sciences Baccalaureate program premedical students’ perceived sleep quality by their actual global Pittsburg Sleep Quality Index score categories.

Pittsburg Sleep Quality Index score (perceived sleep quality)^a^	Optimal (<5)	Slightly poor (5-7)	Borderline poor (8-10)	Poor (11+)
No problem at all (total score=28)	14 (13.6)	9 (8.7)^b^	4 (3.9)^b^	1 (1)^b^
Only a very slight problem (total score=43)	11 (10.7)^b^	21 (20.4)	9 (8.7)^b^	2 (2)^b^
Somewhat of a problem (total score=28)	1 (1)^b^	13 (12.6)^b^	7 (6.8)	7 (7)^b^
A very big problem (total score=4)	0 (0)	0 (0)	1 (1)^b^	3 (3)

^a^0-4 (<5)=optimal sleep; 5-7=slightly poor; 8-10=borderline poor; 11+=poor with overall percentages.

^b^Inconsistency between the perceived sleep quality and Pittsburg Sleep Quality Index scores.

**Table 3:** Significance values of post hoc Dwass-Steel-Critchlow-Fligner multiple comparisons between Medical Sciences Baccalaureate program premedical students’ perceived stress effects.

Perceived stress effect	A very big problem	No problem at all	Only a very slight problem
No problem at all	.048^a^	N/A^b^	N/A
Only a very slight problem	<.001^a^	.99	N/A
Somewhat of a problem	.42	.15	.001^a^

^a^Significant at *P*<.05.

^b^N/A: not applicable.

### Process Evaluation and Participant Feedback

Overall, the participants reported the Fitbit app as a helpful tool in understanding their sleep. They were especially excited to visualize their sleep stage breakdown and obtain an overview of their sleep by simply looking at their Fitbit sleep score. The participants’ feedback on the study protocol was summarized in the following 4 areas.

First, all assessments were conducted in the expected duration, that is, 30 minutes for the preassessment and midassessment and 60 minutes for the postassessment. The web-based mode of midassessments and postassessments did not create any barriers to the data collection. The feedback also revealed that the interview component in all 3 assessments was effective and served different purposes. Specifically, the interview in the preassessment made the participants think about their previous experiences regarding sleep and patient-generated health data devices. The midassessment interview, which reviewed daily reflections at a glance, invoked awareness among participants. The interview in the postassessment was the most comprehensive as it gathered thoughts on daily reflections and assessed Fitbit data comprehension. The administered questionnaires in preassessments, midassessments, and postassessments, as shown in Figure 1, had a 100% response rate because participants were required to complete it during the semistructured interview. The global PSQI scores for 5 participants across the 2 weeks are shown in Figure S1 in Multimedia Appendix 2. The daily reflections also had a 100% response rate, as several reminder emails were sent out to ensure data collection.

Second, the participants did not have a strong opinion about the frequency of the well-being survey administration (3 times over a 2-week period in this study). However, the survey scores did not display much variation or clear trends. Although this can be due to the small group of participants (n=5), it may be more ideal to conduct the survey with more spaced durations.

Third, the Fitbit data provided notable variation daily and was able to grasp all 14 days well for the most part. Some participants experienced cases during which Fitbit would sense bed and wake time but would not be able to provide detailed analytics on the sleep stages or on the sleep score. For these participants, some of the issues included wearing the Fitbit too snug or loose or wearing the Fitbit only right before going to bed. In other cases, participants simply appeared to forget to put on their Fitbit watch after removing it for charging, leading to a void of data points. The participants were fairly adherent to completing daily reflections on time and found that a daily email reminder from the research team helped greatly with this task.

Lastly, the participants identified that the Fitbit app tends to provide vague descriptions. For example, the biggest concern raised was not being able to understand how the sleep score was calculated. This made participants question the score’s reliability and accuracy. Similarly, some participants highlighted redundancy of information (sleep stage condensed cycling), whereas others suggested presenting information in a more visually appealing manner (pie chart to see sleep stage breakdown), individualized manner (suggesting sleep recommendations based on score trends), and sleep score explainability (being specific as to why a score may have dropped/raised on a day-by-day basis).

Finally, the degree to which Fitbit data aligns with the PSQI self-reported data obtained through surveys was assessed. Overall, across the 2-week period, there was a moderately negative correlation (*r*=–0.598) calculated from the data shown in Table S1 of Multimedia Appendix 2. In context, this means high Fitbit scores, indicating good sleep, were correlated with low global PSQI scores, also indicating good quality of sleep. Week 1 had a strong negative correlation (*r*=–0.87) compared to Week 2, which had a moderate negative correlation (*r*=–0.648).

## Discussion

### Principal Findings

This study presents the sleep patterns of premedical students through a sleep and well-being survey and evaluates a study protocol in preparation for a large-scale study. The survey showed that although the participants did have an average of 7 hours of sleep in general, only a quarter of them had good sleep quality. There was no difference in sleep quality among the student cohorts. Furthermore, there was an inconsistency between the perceived sleep quality by the students versus what their PSQI scores reflected. The poor sleep quality experienced by unaware students points to the need for self-monitoring through wearables such as Fitbit devices to increase sleep quality awareness. Lastly, a comparison of stress effect on sleep with PSQI scores establishes that sleep is not the only dominant factor in PSQI. The participants who perceived stress to play no role in their sleep still displayed a wide range of PSQI scores. Our findings show that the study protocol is highly feasible in terms of assessment durations and interview effectiveness. The usage of Fitbit devices did increase participants’ awareness regarding their sleep based on the qualitative feedback, along with their overall understanding of sleep factors. Participants keeping track of their sleep via daily reflections also aided in their cognizance. Based on the feedback, the study protocol can be improved by extending the duration, educating individuals about the Fitbit-wearing behaviors, and increasing the sleep score interpretability.

In assessing how the Fitbit sleep score data align with the global PSQI scores, we found a moderate negative correlation. This makes sense because higher Fitbit scores, suggesting good sleep, aligned with lower global PSQI scores, which indicates good sleep quality. Therefore, achieving moderate to high correlation displays a good potential for Fitbit sleep scores to serve as a proxy for PSQI global scores. Although week 1 gathered a stronger correlation than week 2, we believe that this result may be due to greater missing data in week 2, either from fatigue or nonadherence.

### Implications

Our study shows that only 25.2% (26/103) of the participating premedical students had good sleep quality, as indicated by the PSQI scores. This proportion is lower than that (34.1%) previously reported among college students in general [10]. This leads us to a possible inference that premedical students may have sleep patterns different from those of college students overall. Our study also introduces a new direction, in which sleep patterns are not only measured but further analyzed to determine discrepancies between perception versus actual sleep quality. Prior studies [17,46] looking at sleep patterns in medical school students or undergraduate college students in general could also benefit from this approach and take their sleep analysis to the next step. For example, a 2016 study conducted on undergraduates at the University of California explored relations between sleep and mental health in individuals with healthy sleep habits [17]. Hence, further understanding the sleep perceptions of these healthy students could be used to create a benchmark to which other types of sleepers can be measured relatively. Another application could include a longitudinal study, similar to the one proposed by the University of South Wales in Australia [46]. This 5-year study provides insights into the mental health of medical students, with sleep being one of the factors measured via Fitbit. By incorporating the concept of sleep and well-being perceptions, the longitudinal approach may provide a better understanding of whether perceptions stay consistent or change over time.

The increase in awareness regarding sleep due to Fitbit usage mentioned by the participants also opens an area for possible interventions. As previous literature has reported, when individuals are educated in a personally relevant manner, their awareness leads to behavior change [47-49]. An important component of behavior change is self-efficacy, which is the perception of one’s ability to achieve certain outcomes [50-52]. Hence, interventions can begin with Fitbit usage to increase participant awareness regarding why they need to make a change, establish how certain health behaviors will lead to expected outcomes, and then provide ways for participants to reach those outcomes [53,54]. Conversely, 1 study has tested a habit loop model, which is based on cue, routine, and reward [55]. The Fitbit usage behavior in terms of the habit loop model can be as follows: trust in Fitbit accuracy of physical and sleep data (cue), intensity of Fitbit use (routine), and adjustment of physical and sleep behaviors (reward). Although this study shows that trust in Fitbit produced little changes in sleep and health behaviors, future studies should be built upon this foundation to examine the potential behavioral change through the supplementation of educational information to participants.

In the evaluation of the protocol itself, there were inconsistencies in obtaining sleep scores from Fitbit at times. A pre-existing measure during this study was placing instructions in the daily reflection reminder email to contact the research team immediately if they received no score in order for the research team to troubleshoot. Some other troubleshooting measures could help ensure participants wear their Fitbit at all times. For example, the study team can propose a standardized charging time that prompts participants to remove and charge their Fitbits during our week 1 midassessment session and wear the Fitbit again at the conclusion of the meeting. This can prevent participants from forgetting to wear their Fitbits during the study period. Not all participants explored all the features of Fitbit’s sleep interface. This points to the need to give participants a tutorial about the various types of analytical information available that participants can browse through to understand their sleep patterns. Finally, for the Fitbit data retrieval process, researchers asked the participants to download their data from the Fitbit.com website in week 1 and week 2 assessments. This proved to be a time-consuming process; therefore, the protocol was updated to begin data download at the beginning of the assessment meeting, which allowed the download to be finished by the end of the meeting. These lessons learned help to refine our study protocol and will lead to higher data quality in our large-scale study.

### Limitations

This study is limited in several ways. First, the assessment was conducted with premedical students from a single program in a single institution. Hence, it is possible that the findings were limited to the student demographics in our institution. Additionally, the well-being survey administered in this study did not ask for their sex, race, or age due to the constraints in the institutional review board protocol. Thus, this analysis does not demonstrate whether demographic factors are linked with certain sleep patterns. Moreover, this study did not consider the academic performance of the participants and its relationship with sleep patterns. Demographic information and academic performance will be collected in our future studies with an updated institutional review board protocol. Next, we only recruited 5 students via convenience sampling for protocol evaluation. Small sample sizes introduce the possibility of skewed opinions. However, it should be noted that the goal of this study was to test the protocol rather than any hypothesis. Additionally, the Fitbit usage habits demonstrated by the pilot study group point to the need to improve the Fitbit usage protocol. For example, students were advised to always wear their Fitbits except while charging. However, some students after charging their Fitbit forgot to wear it overnight, thereby leading to nonadherence. Another instance involved a participant using the “sleep now” function offered by the watch to track sleep, even though the participants were not instructed on that feature’s use. Using that function did dysregulate the visualization data, which returned to normal once the participant was asked to terminate the function's usage.

### Future Work

With the findings gathered from this pilot study, we hope to expand to a larger experiment within the MSBP and expand to premedical programs at other institutions. We hope to address sleep quality discrepancies and determine a feasible solution for sleep hygiene improvement in premedical students. Another future work involves the calculation of Fitbit sleep scores especially when the sleep data are collected but the sleep score is missing. In this study, we tried to explore how the Fitbit score was calculated and attempted to assess trends. Fitbit informs everyone that their sleep score is devised from 3 pieces of information: time asleep and awake, deep and rapid eye movement sleep, and sleeping heart rate and restlessness. However, it is unclear how exactly the sleep score values are formed. Therefore, it was difficult to generate actionable information. Hence, this topic should be further explored to determine whether sleep scores are indeed valid measures of sleep.

### Conclusions

We conducted a pilot study to understand the sleep patterns of premedical undergraduate students and evaluated the study protocol. The survey results showed a generally lower sleep quality for this student population and posited a gap between the perceived sleep quality versus PSQI-measured sleep quality. This gap may be solved by the awareness raised by Fitbit usage. We will revise the study protocol based on the lessons learned and health behavior theories and conduct a large-scale experiment to use Fitbit as an informatics solution to promote healthy sleep behaviors.

## Appendix

Multimedia Appendix 1 Survey data.

Multimedia Appendix 2 Supplementary data.

## Abbreviations

MSBP: Medical Sciences Baccalaureate Program
PSG: polysomnography
PSQI: Pittsburg Sleep Quality Index
RQ: research question

## Data Availability

All data generated or analyzed during this study are included in this paper and multimedia appendices.
