# Supplementary material for: Sleep Patterns of Premedical Undergraduate Students: Pilot Study and Protocol Evaluation
Source: JMIR Form Res. 2024 Feb 2;8:e45910. doi: 10.2196/45910 (PMC10873796; doi:10.2196/45910)
Supplement: Multimedia Appendix 2 [file formative_v8i1e45910_app2.docx]

**Supplemental Figure 1.** Line graph displaying Global PSQI scores of Participants 01(blue), 02(orange), 03(grey), 04(yellow), and 05(light blue) across the three assessments periods (Week 0, Week 1, Week 2) during the 2-week pilot study. The Global PSQI scores were calculated by using the Pittsburgh Sleep Quality Index questionnaire. The response rate for the survey was 100% because it was conducted during the mandatory semi-structured assessment meeting.


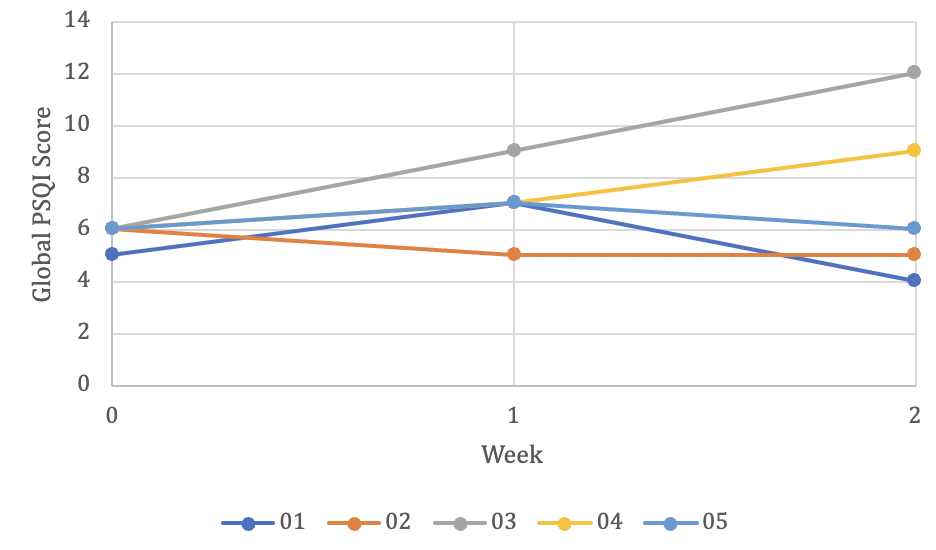


**Supplemental Table 1.** Below are the average global PSQI scores as well as Fitbit Sleep scores for participants 1-5 across week 1 and week 2. The sleep score count refers to how many sleep scores were detected by Fitbit for the corresponding week. The PSQI average and sleep score average data was used in the calculation of the Pearson correlation coefficients to assess how Fitbit data aligns with self-reported survey data. Across the two-week period, there was a moderately negative correlation (r= -0.598) calculated from the data shown in Supplemental Table 1. In context, this means higher Fitbit scores, indicating good sleep, were correlated with low global PSQI scores, also indicating good quality of sleep. Week 1 had a strong negative correlation (r= -0.87) compared to Week 2 which had a moderate negative correlation (r= -0.648).

| PID | Week # | PSQI Average | Sleep Score Count | Sleep Score Average |
| --- | --- | --- | --- | --- |
| 01 | 1 | 6.0 | 7 | 79.00 |
| 01 | 2 | 5.5 | 7 | 78.49 |
| 02 | 1 | 5.5 | 5 | 81.20 |
| 02 | 2 | 5.0 | 5 | 83.00 |
| 03 | 1 | 7.5 | 6 | 67.50 |
| 03 | 2 | 10.5 | 1 | 74.00 |
| 04 | 1 | 6.5 | 7 | 71.86 |
| 04 | 2 | 8.0 | 3 | 67.33 |
| 05 | 1 | 6.5 | 7 | 79.71 |
| 05 | 2 | 6.5 | 7 | 81.58 |
